# Sublingual apomorphine for Parkinsonian tremor: a post-hoc analysis of pivotal trial data

**DOI:** 10.1007/s00702-026-03225-w

**Published:** 2026-09-08

**Authors:** Haidar S. Dafsari, João Reis, Kerstin Strauß, Helena C. Brigas, Carmen Denecke Muhr, Isabel Pijuan Jiménez, Joerg Holenz

**Affiliations:** 1 Department of Neurology, Faculty of Medicine and University Hospital Cologne, University of Cologne, Kerpener Straße, 50937 Cologne, Germany; 2 BIAL- Portela & Cª, S.A, Porto, Portugal; 3 BIAL Deutschland GmbH, Mörfelden-Walldorf, Germany

**Keywords:** Apomorphine, Motor fluctuations, Parkinson disease, Sublingual, Tremor

## Abstract

Tremor is a common and disabling feature of Parkinson’s disease (PD) and often responds suboptimally to oral dopaminergic therapy. This post‑hoc analysis evaluated tremor outcomes associated with apomorphine sublingual film (SL‑APO) in patients with a tremor‑dominant PD phenotype, using data from a 12‑week randomised, double‑blind, placebo‑controlled trial (NCT02469090) and a 12‑month open‑label study (NCT02542696) conducted in levodopa‑responsive patients with OFF episodes. Tremor‑dominant phenotype was defined according to Movement Disorder Society–Unified Parkinson’s Disease Rating Scale (MDS‑UPDRS) criteria, and tremor severity was assessed using a derived tremor index. In the randomised trial, 31 of 108 patients (28.7%) met tremor‑dominant criteria. At Week 12 (*n* = 10 per group), greater reductions in tremor index scores were observed 30 min post‑dose with SL‑APO versus placebo (mean change − 5.3 vs. − 1.1), with an exploratory between‑group difference of − 4.4 (*p* < 0.001) and an effect size (Cohen’s d) of − 1.83. Clinically important improvement occurred more frequently with SL‑APO (80%) than placebo (30%), corresponding to an estimated number needed to treat of 1.3. Median time to ON with SL‑APO was 13.0 min. In the open‑label study, tremor reductions of 31–37% were maintained over 12 months, with responder rates remaining above 70%. These exploratory findings in a small subgroup are consistent with a potential for early and maintained tremor improvement with SL‑APO in patients with tremor‑dominant PD; however, the results are hypothesis‑generating and require prospective confirmation.

**Supplementary Information:**

The online version contains supplementary material available at https://doi.org/10.1007/s00702-026-03225-w.

## Introduction

Tremor is one of the most visible and burdensome manifestations of Parkinson’s disease (PD) and is often among the earliest symptoms to emerge (Pasquini et al. 2023). Although classically described as a 4–6 Hz resting tremor, many patients experience multiple tremor subtypes (rest, postural, and kinetic), each of which can independently impair everyday functioning. Despite its prominence across the disease course and strong association with diminished quality of life and stigma, treatment options remain limited (Pasquini et al. 2023; Hauser et al. 2025; Angulo et al. 2019). Moreover, tremor may persist or re‑emerge during OFF periods even in patients with otherwise adequate control of bradykinesia and rigidity, highlighting an unmet need for therapies that can provide rapid and reliable symptom relief (Hauser et al. 2025). This variability in dopaminergic responsiveness further underscores the need for fast‑acting treatment strategies specifically targeting tremor fluctuations.

Apomorphine, a potent non‑ergot dopamine agonist with a broad‑spectrum receptor profile closely resembling that of dopamine, offers rapid and robust dopaminergic stimulation (Jenner and Katzenschlager 2016; Yegla et al. 2025). Its ability to improve motor symptoms is regarded as comparable to levodopa, and its rapid onset combined with a short clearance half‑life makes it well suited for on‑demand use to self‑manage OFF episodes and return to the ON state quickly and reliably (Jenner and Katzenschlager 2016). Apomorphine sublingual film (SL‑APO) was developed to provide a fast‑acting, easy‑to‑administer alternative to injections (Kassubek et al. 2024b) supported by patient preference evidence showing sublingual delivery is favoured over inhaled and injected routes regardless of adverse events (Thach et al. 2021) and preferred over the subcutaneous apomorphine pen with higher satisfaction despite comparable safety in a clinical study (Stocchi et al. 2023). In pivotal clinical trials, treatment with SL-APO was efficacious within 15 min following administration, with peak effects observed approximately 30 min and sustained for up to 90 min post-intake (Olanow et al. 2020; Kassubek et al. 2024a). Given its pharmacokinetic profile, delivering rapid systemic exposure without reliance on gastrointestinal absorption, SL‑APO may be particularly well suited to address symptoms that are sensitive to delayed or fluctuating levodopa absorption. Such properties are of particular relevance in the context of tremor, where both delayed onset and inconsistent response to oral therapy are frequently reported (Hauser et al. 2025).

A single centre retrospective review of patients with task-specific tremor who later developed PD reported improvement in levodopa-refractory tremor with SL‑APO in 6/8 patients (Ondo et al. 2023). Given that parkinsonian resting tremor often responds poorly to oral dopaminergic therapy (Pasquini et al. 2023; Hauser et al. 2025), we hypothesized that SL‑APO could represent an effective treatment option. This *post‑hoc* analysis of pivotal trial data examined the effects of SL‑APO on parkinsonian tremor over 12 weeks and 12 months in PD patients with a tremor‑dominant phenotype.

## Methods

Full methodological details of the pivotal 12-week, randomized, placebo-controlled trial (RCT; NCT02469090) (Olanow et al. 2020) and open-label, single-arm, 12-month study (NCT02542696) (Kassubek et al. 2024a) of SL-APO in patients with OFF episodes have been previously published. Both trials were approved by institutional review boards at participating sites, conducted in accordance with the Declaration of Helsinki and International Council for Harmonization Good Clinical Practice guidelines, and included written informed consent from all patients prior to study procedures.

Briefly, both multicentre studies enrolled levodopa‑responsive patients with PD on stable background antiparkinsonian therapy who continued to experience predictable OFF periods (≥ 2 h/day), including early morning OFF. The RCT included a levodopa challenge test during screening, with Movement Disorder Society‑Unified Parkinson’s Disease Rating Scale (MDS‑UPDRS) Part III scores assessed before and after the usual morning levodopa/carbidopa dose without adjunctive PD medication (Olanow et al. 2020; Hui et al. 2020). Both studies incorporated individualized dose titration to establish an effective dose. Once titrated, patients could self‑administer SL‑APO for up to 5 OFF episodes per day, with ≥ 2 h between doses. Motor function (MDS‑UPDRS and motor state) was assessed at 15, 30, 45 (RCT only), 60 and 90 min post‑intake. In the RCT, the primary endpoint was change in MDS‑UPDRS motor score at 30 min post‑intake at Week 12 (Olanow et al. 2020); motor assessment was a secondary outcome in the open‑label study (Kassubek et al. 2024a). Time‑to‑ON in the RCT was defined as the first timepoint at which patients reached full ON, comparable to their levodopa response (Olanow et al. 2020).

For this *post‑hoc* analysis, tremor‑dominant PD was defined according to Stebbins et al. (2013) using the pre‑dose MDS‑UPDRS tremor (items 2.10 and 3.15–3.18) and postural instability/gait difficulty (PIGD) items (items 2.12–2.13 and 3.10–3.12). Mean tremor and PIGD scores were calculated, and patients with a tremor‑to‑PIGD ratio ≥ 1.15 were classified as tremor dominant (Stebbins et al. 2013). Tremor efficacy was evaluated using the tremor index, defined as the sum of tremor‑related MDS‑UPDRS items, assessed at 30 min post‑intake at Week 12 of the RCT and at months 3, 6 and 12 of the open‑label study.

The inclusion of both double‑blind and open‑label data enables assessment of both short‑term treatment effects and the durability of response under conditions reflective of routine clinical use. Data were summarized descriptively. Scores at 30 min post‑intake were compared with pre‑intake values using paired t tests (*p* < 0.05, two‑sided). Between‑group differences were analysed using ANCOVA with treatment arm as a fixed factor and pre‑intake tremor score as a covariate; effect sizes were calculated using Cohen’s d (Cohen). Percent tremor reduction was normalized to the maximum tremor index score of 20. Responders were defined as patients achieving at least the minimal clinically important change (MIC), calculated as one‑half the pre‑dose tremor score SD (Norman et al. 2003). The number needed to treat (NNT) was calculated as (1/% responders) × 100. Treatment effects across open‑label timepoints were assessed using a linear mixed‑effects model with baseline as a fixed covariate and subject as a random effect. No imputation was performed for missing data. Univariate logistic regression was used to compare responders and non‑responders.

## Results

**Fig. 1:**
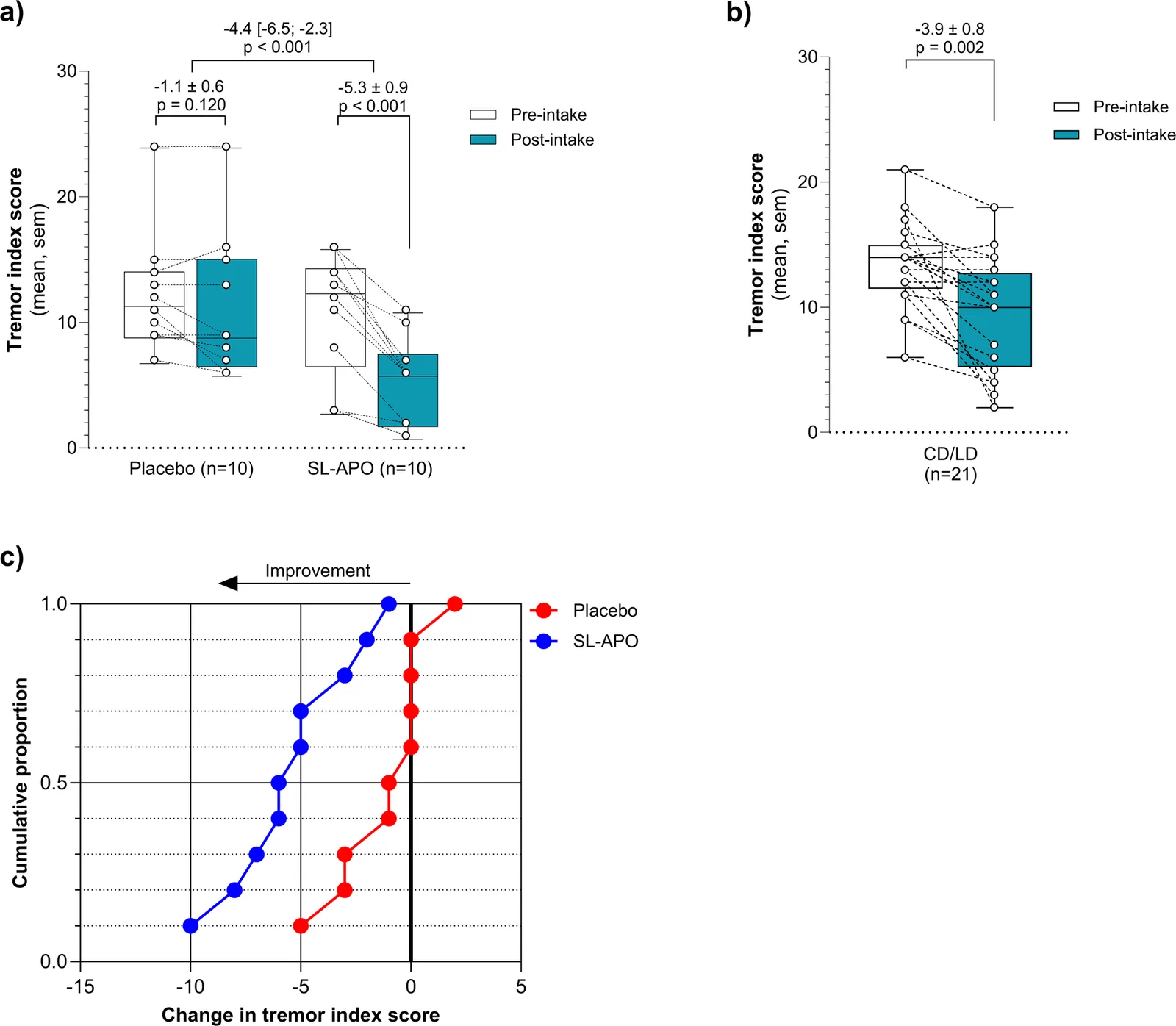
Tremor index scores: (**a**) from pre-intake to 30 min post-intake at Week 12 of the randomized controlled trial*; (**b**) during the levodopa/carbidopa challenge test at screening; and (**c**) cumulative distribution curve. *Pre- and post-dose intake data were available for 20 patients

Of the 109 patients in the RCT intention‑to‑treat population, 31/108 (28.7%) were classified *post-hoc* as tremor‑dominant, of whom 21 completed the study and 10 early discontinued. Seventeen patients (55%) were randomised to receive SL‑APO and 14 (45%) placebo; baseline tremor scores were balanced (mean ± SD: SL‑APO 13.3 ± 5.2; placebo 12.8 ± 3.6). As expected, participants who did not meet tremor-dominant criteria had a lower mean baseline tremor score (5.3 ± 5.0; *p* < 0.001 versus the tremor-dominant subgroup) (Fig. 1).

After 12 weeks, SL‑APO provided greater tremor improvement than placebo in the tremor-dominant population. Mean ± SD tremor index scores decreased from 11.0 ± 4.8 pre‑dose to 5.7 ± 3.3 at 30 min post‑intake with SL‑APO (*n* = 10), versus 12.4 ± 4.8 to 11.3 ± 5.7 with placebo (*n* = 10). The within‑group change was − 5.3 [− 7.3; −3.3] with SL‑APO and − 1.1 [− 2.5; 0.3] with placebo, yielding a between‑group treatment effect of − 4.4 [− 6.5; −2.3] (*p* < 0.001; Fig. 1a) and a large effect size (Cohen’s d = − 1.83). Improvements reflected reductions in both rest and postural tremor. At 30 min, adjusted tremor reductions were 27% with SL-APO and 6% with placebo, and the mean reduction with SL-APO exceeded that observed during the levodopa challenge (− 5.3 ± 2.8 with SL‑APO at Week 12 vs. − 3.9 ± 3.7; Fig. 1b). Based on a calculated MIC of 2.3, responder rates were 80% with SL‑APO and 30% with placebo, supported by cumulative distribution function plots showing a consistent shift toward greater improvement with SL‑APO (Fig. 1c). The NNT was 1.3 for SL‑APO and 3.3 for placebo. Median [range] time‑to‑ON with SL‑APO at Week 12 was 13.0 [3.2–29.1] minutes.

**Fig. 2:**
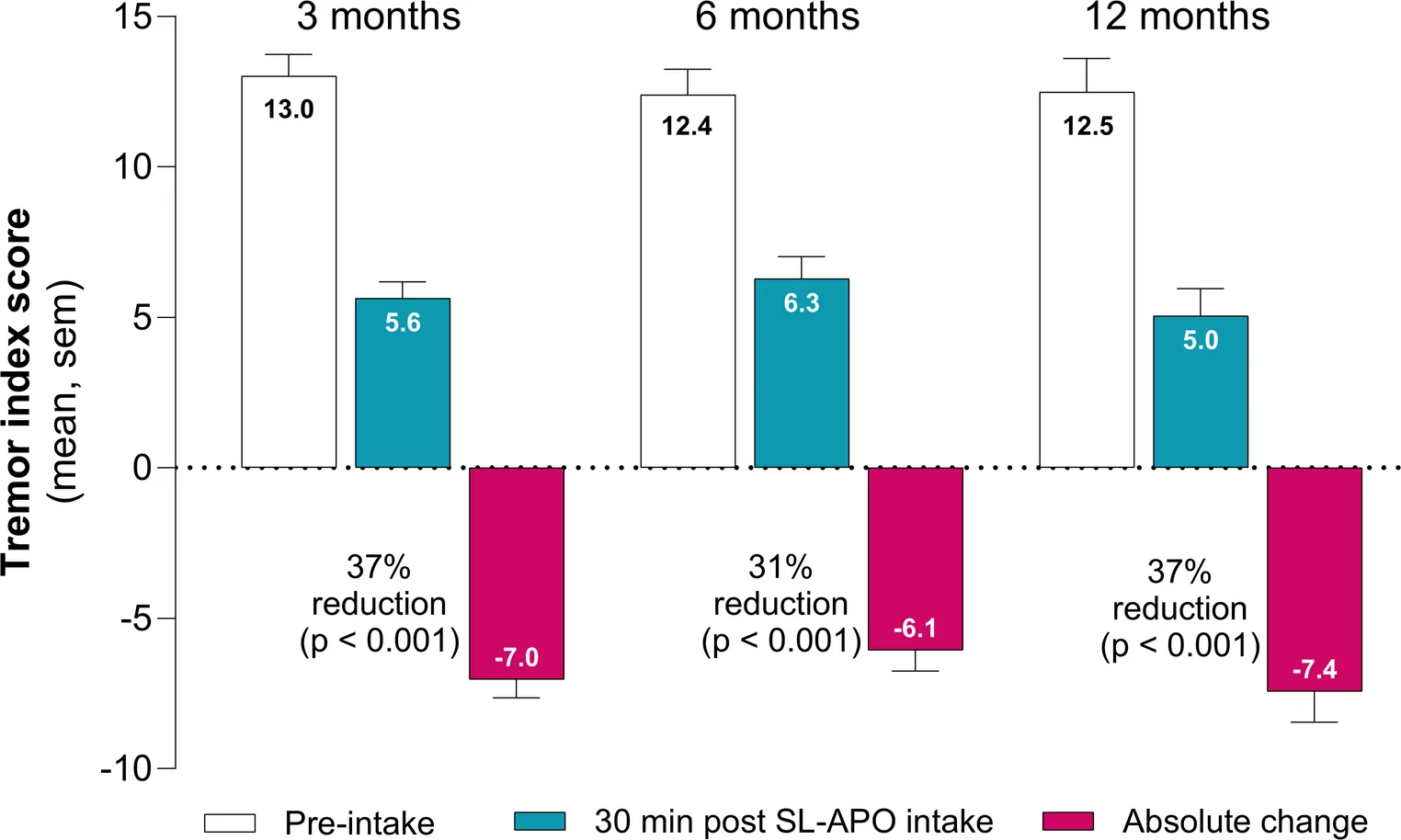
Tremor index scores at months 3, 6, and 12 of the open-label study

In the open-label study, 64/302 patients (21.2%) were tremor dominant and pre-intake data were available for 64 patients at 3 months, 51 (79.7%) at 6 months, and 23 (35.9%) at 12 months. SL‑APO produced sustained tremor index reductions of 37% (− 7.03 points) at month 3, 31% (− 6.1 points) at month 6, and 37% (− 7.4 points) at month 12, with no significant differences across timepoints. For patients with post-intake data, using a baseline MIC of 2.9, responder rates remained stable at 75.0% (45/60), 76.0% (38/50), and 73.9% (17/23) at months 3, 6, and 12, respectively (Fig. 2).

Logistic regression analyses showed no significant differences between responders and non‑responders across demographic or disease‑related variables, including country, age, sex, Hoehn & Yahr stage, disease duration, duration or type of OFF episodes, number of daily OFF episodes, and concomitant anti-PD medication (i.e. amantadine, dopamine agonists, COMT inhibitor or MAO-B inhibitor use).

## Discussion

Tremor is often the most difficult motor symptom of PD to manage, with many patients experiencing insufficient relief even when other dopaminergic symptoms respond well to treatment (Pasquini et al. 2023; Hauser et al. 2025). This *post‑hoc* analysis of pivotal trial data shows that SL‑APO produces rapid, clinically important tremor reductions in patients with tremor‑dominant PD and motor fluctuations, supporting further evaluation of its potential as an on-demand therapy during OFF episodes. From a clinical perspective, the rapid onset of effect is particularly relevant, as tremor occurring during OFF periods can be both distressing and functionally limiting, often requiring prompt control.

In the RCT, 12 weeks of SL‑APO treatment resulted in greater tremor reductions than placebo, with a large effect size and high responder rate. The magnitude of improvement, in both absolute tremor index reduction and percentage change, is notable given the pharmacological challenges of treating tremor (Pasquini et al. 2023). This effect size should be interpreted in the context of the enriched tremor‑dominant subgroup analysed here, as baseline tremor severity in the overall study population was substantially lower; such a lower tremor burden would be expected to dilute measurable treatment effects, such that potential benefits of SL‑APO on tremor may remain undetectable outside this subgroup. Consistent with this, the rapid onset of benefit, with a median time‑to‑ON of 13.0 min, was faster than in the full study population (21.2 min) (Olanow et al. 2020), despite tremor typically showing delayed dopaminergic responsiveness relative to rigidity or bradykinesia due to involvement of non–basal ganglia circuitry (Rodriguez-Oroz et al. 2009). Given that time‑to‑ON reflects attainment of the overall motor ON state rather than a tremor‑specific endpoint, it would be expected to incorporate tremor control as part of the broader motor response.

Consistent with previously reported superior overall MDS‑UPDRS motor effects (Hui et al. 2020), the improvement seen with SL‑APO at 30 min exceeded that observed during the levodopa challenge at screening, suggesting a more robust dopaminergic effect on tremor in this subgroup. The rapid pharmacodynamic profile of SL‑APO (Pappert et al. 2016; Hauser et al. 2016; Olanow et al. 2020; Kassubek et al. 2024a) may allow more effective engagement of dopaminergic pathways at the time of symptom re‑emergence. Additionally, although the mechanism is unclear, emerging data indicate that apomorphine engages dopamine receptor cAMP and β‑arrestin signalling pathways differently from dopamine, and such differences in secondary messenger system activation may help explain the observed effect (Yegla et al. 2025).

The long-term open-label data further support the durability of SL-APO’s tremor-reducing effects. Across 12 months, reductions in tremor index scores were maintained, with responder rates consistently above 70%. This sustained effect is relevant as it suggests that repeated intermittent dosing may not be associated with an obvious loss of efficacy over time—an important consideration for therapies intended for long-term, as-needed use. Logistic regression analyses showed no clear demographic or disease‑related predictors of tremor response, with efficacy appearing independent of age, sex, disease duration, Hoehn & Yahr stage, OFF‑episode characteristics or concomitant PD medication, suggesting potential applicability of SL‑APO across a tremor‑dominant population. However, these findings should be interpreted cautiously given the exploratory nature of the analyses and the possibility of attrition bias inherent to long‑term open‑label studies where patients with poorer response are more likely to discontinue. Discontinuation rates at 6 and 12 months were broadly consistent with the parent open-label trial, in which the most common reasons for early withdrawal were adverse events and withdrawal of consent (Kassubek et al. 2024a). Although no clear predictors of response were identified, the limited sample size and potential for selective retention preclude definitive conclusions. Nevertheless, the absence of an obvious demographic or disease‑related signal suggests that further prospective investigation in broader populations may be warranted. Although the present analysis specifically evaluated SL-APO, the observed findings may support further investigation on the effects of other rapidly acting apomorphine-based therapies, such as subcutaneous apomorphine.

Several additional limitations should be acknowledged. The RCT enrolled only patients who achieved full ON with SL‑APO during titration (relative to the levodopa response), thereby enriching the population for treatment responders and raising questions about the magnitude of efficacy in an unselected population. However, as noted by Olanow et al. for the parent population, home-diary data showed that although patients in the active and placebo groups used the study drug to a similar extent in the home setting, the frequency of achieving a full ON response strongly favoured SL-APO (Olanow et al. 2020). Tremor‑dominant patients comprised a relatively small proportion of the RCT cohort (28.7%), compared with up to 44% in clinical practice (Konno et al. 2018) and 68% in early disease (Lee et al. 2019), likely reflecting the advanced disease stage of the enrolled population (typically 9 years since diagnosis (Olanow et al. 2020) and the known shift toward a PIGD phenotype with disease progression (Lee et al. 2019). Consequently, our subgroup analyses were limited to small patient numbers. In addition, the tremor index, while derived from validated MDS‑UPDRS items, may not fully capture all aspects of tremor severity, including amplitude variability or functional impact. As these post‑hoc analyses were not powered for tremor‑specific outcomes nor adjusted for multiplicity, the findings should be interpreted in a hypothesis‑generating context.

## Conclusions

To the best of our knowledge, we report the first analyses evaluating the effects of SL‑APO on tremor in patients with a tremor‑predominant phenotype. Taken together, these findings are consistent with the concept that, despite partial resistance to oral dopaminergic therapy, PD tremor may remain substantially dopaminergic in many patients. The rapid and potent receptor stimulation achieved with SL‑APO may partially offset pharmacokinetic limitations of oral levodopa, such as delayed gastric emptying and fluctuating plasma levels, potentially supporting more consistent tremor control. Prospective evaluation, preferably including tremor‑specific validated measures or actigraphy, is required to confirm these exploratory, hypothesis‑generating observations, which nonetheless suggest a potential role for SL‑APO in the management of OFF‑related parkinsonian tremor.

## Supplementary Information

Below is the link to the electronic supplementary material.

Figure e1 Baseline tremor index score for tremor dominant and non-tremor dominant phenotypes within the total, placebo and sublingual apomorphine population

## Data Availability

All available post-hoc data are contained within the publication.
